# Autophagy-related cell death by pan-histone deacetylase inhibition in liver cancer

**DOI:** 10.18632/oncotarget.8585

**Published:** 2016-04-05

**Authors:** Pietro Di Fazio, Petra Waldegger, Samir Jabari, Susanne Lingelbach, Roberta Montalbano, Matthias Ocker, Emily P. Slater, Detlef K. Bartsch, Romana Illig, Daniel Neureiter, Thaddeus T. Wissniowski

**Affiliations:** ^1^ Department of Visceral, Thoracic and Vascular Surgery, Philipps University of Marburg, Marburg, Germany; ^2^ Institute for Biomedical Aging Research, University of Innsbruck, Rennweg, Innsbruck, Austria; ^3^ Institute for Anatomy I, University of Erlangen-Nurnberg, Erlangen, Germany; ^4^ Department of Urology, Philipps University of Marburg, Marburg, Germany; ^5^ Institute for Surgical Research, Philipps University of Marburg, Marburg, Germany; ^6^ Institute of Pathology, Paracelsus Medical University/Salzburger Landeskliniken (SALK), Salzburg, Austria; ^7^ Department of Gastroenterology and Endocrinology, Philipps University of Marburg, Marburg, Germany; ^8^ Experimental Medicine Oncology, Bayer Pharma AG, Berlin Germany

**Keywords:** autophagic cell death, liver cancer, pan-deacetylase inhibitor, cancer therapy, autophagosomes

## Abstract

Autophagy is a homeostatic, catabolic degradation process and cell fate essential regulatory mechanism. Protracted autophagy triggers cell death; its aberrant function is responsible for several malignancies. Panobinostat, a potent pan-deacetylase inhibitor, causes endoplasmic reticulum stress-induced cell death. The aim of this study was to investigate the role of autophagy in deacetylase inhibitor-triggered liver cancer cell death.

HepG2 (p53wt) and Hep3B (p53 null) liver cancer cell lines were exposed to panobinostat. RT-qPCR and western blot confirmed autophagic factor modulation. Immuno-fluorescence, -precipitation and -histochemistry as well as transmission electron microscopy verified autophagosome formation. The cytotoxicity of panobinostat and autophagy modulators was detected using a real time cell viability assay.

Panobinostat induced autophagy-related factor expression and aggregation. Map1LC3B and Beclin1 were significantly over-expressed in HepG2 xenografts in nude mice treated with panobinostat for 4 weeks. Subcellular distribution of Beclin1 increased with the appearance of autophagosomes-like aggregates. Cytosolic loss of p53, in HepG2, and p73, in Hep3B cells, and a corresponding gain of their nuclear level, together with modulation of DRAM1, were observed. Autophagosome aggregation was visible after 6 h of treatment. Treatment of cells stably expressing GFP-RFPtag Map1LC3B resulted in aggregation and a fluorescence switch, thus confirming autophagosome formation and maturation. Tamoxifen, an inducer of autophagy, caused only a block in cell proliferation; but in combination with panobinostat it resulted in cell death.

Autophagy triggers cell demise in liver cancer. Its modulation by the combination of tamoxifen and panobinostat could be a new option for palliative treatment of hepatocellular carcinoma.

## INTRODUCTION

Hepatocellular carcinoma (HCC) represents the third most common cause of cancer-related death worldwide. It has a poor prognosis and the current therapy has a poor outcome. Even with treatment, such as transarterial chemoembolization (TACE), intraarterial chemoinfusion, systemic chemotherapy, radiotherapy, immunotherapy or hormonal therapy, the 5-year relative survival rate for patients with HCC is only 7% [1]. The paucity of effective and well-tolerated treatments for advanced HCC highlights the need for new therapeutic approaches [2].

Autophagy is a catabolic process that prevents the accumulation of damaged proteins and organelles within cells [3]. Autophagy represents a double-edged sword due to its dual role as tumor suppressor resulting in the degradation of metabolic and structural proteins and subcellular organelles as well as enabler of cell survival that facilitates tumor growth [4]. The process was first described in S. cerevisiae, where it possesses a survival property only [5]. To date, autophagy has been described in all vertebrates, including humans. Autophagy impairment, caused by loss of function of autophagy (Atg) genes, has been associated with aging, heart and muscle degeneration, neurodegeneration and cancer. This catabolic mechanism is a well-regulated process characterized by several checkpoints. The alteration of any of these checkpoints causes an impairment of the autophagic mechanism leading to pathological side effects. An impaired recycle system, characterized by the loss of autophagosome and lysosome fusion, causes a massive accumulation of metabolites leading to cellular stress and genetic instability leading to tumorigenesis [4]. Nonetheless, alteration of genes attributed to the initial autophagosomes formation, e.g. Beclin1 (Atg6), Atg5, Atg12, UVRAG (UV radiation resistance-associated gene) and MAP1LC3 (Microtubule-associated proteins 1A/1B light chain 3), has been linked to tumor development [6, 7]. Beclin1^+/−^ mice spontaneously develop liver and lung tumors when expressing the large envelope HBV protein [8]. Alteration of AMPK (5ʹ AMP-activated protein kinase) after treatment with Sorafenib in HCC cells resulted in a distinct activation of autophagy [9]. Additionally, mono-allelic deletion of UVRAG is associated with colon cancer progression [10] and altered expression of MAP1LC3 and LAMP2 (Lysosome-associated membrane protein 2), a protein responsible for the final fusion of the autophagosome with lysosomes, is implicated in the development of neuronal and heart diseases as well as cancer [11]. Mutation of SQSTM1 is linked to Paget's disease by causing abnormal bone turnover that results in arthritis, bone deformation and nerve injury [12].

Autophagy is normally executed at basal level in every cell and promotes cellular homeostasis and organelle turnover [13]. Under certain stress conditions, autophagy also can promote cell death [14]. Cancer cells require the autophagic mechanism in order to overcome certain stress conditions such as nutrient deprivation and ROS (Reactive Oxygen Species) production via degradation of non-functional organelles and metabolites, which, in turn, results in metabolite self-recruitment. Moreover, a protracted autophagic mechanism can trigger cell death and represents a key mechanism for the fate of cancer cells [15].

DACi are currently being investigated in clinical trials for the treatment of solid and hematologic malignancies. In particular, Zolinza (Vorinostat, SAHA) and FK228 (Romidepsin) already have been approved by the FDA for the treatment of cutaneous T-cell lymphoma [16]. These DACi and others, such as panobinostat, have stimulated great interest due to their ability to induce cell demise in cancer cells. The mechanism of action is related not only to their ability to block HDACs and promote chromatin hyperacetylation, but also to modulate the expression and the activity of nuclear and cytosolic key players in cell fate, e.g. TP53, HSP90 and miRNAs [17].

Panobinostat, in particular, already has been shown to promote cell death in a P53-independent manner, via induction of alternative apoptotic cell death mediated by endoplasmic reticulum (ER)-stress [18, 19]. Additionally, panobinostat was found to induce the expression of the tumor suppressor hsa-let-7b and to prevent the expression of the oncogenic miR17-92 miRNAs cluster in liver cancer cells. This leads to the repression of the oncogenic transcription co-factor HMGA2 (High-mobility group AT-hook 2) and facilitation of the re-expression of cell death promoter factors like Beclin1 and APAF1 (Apoptotic protease activating factor 1) [20, 21].

This study elucidates the ability of panobinostat to modulate the autophagic mechanism in a cell death *scenario*: the liver cancer cell demise.

The ability of panobinostat to induce cell death *via* autophagy could represent a new aspect of its chemical properties and might aid the current problem of finding a specific treatment for cancer disease, e.g. HCC.

## RESULTS

### Autophagy marker analysis in HCC cells

**Figure 1:**
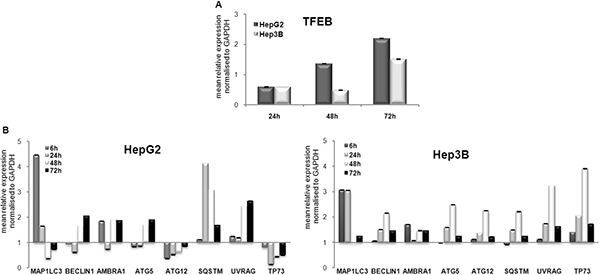
Autophagic marker transcript modulation (**A**) RT-qPCR analysis of TFEB in HepG2 and Hep3B cells after 72 h of treatment with 100 nM panobinostat. (**B**) MAP1LC3, BECLIN1, AMBRA1, ATG5, ATG12, SQSTM, UVRAG, TP73 were analyzed in HepG2 and Hep3B cells after 6, 24, 48 and 72 h incubation with 100 nM panobinostat. mRNA expression was normalized to GAPDH and results are expressed relative to untreated controls set at 1.0. Shown are means ± SEM of three independent experiments performed in triplicates.

Several factors have been identified previously as specific autophagy markers [22]. The first step in this study focused on the analysis of the expression of the autophagy-modulating transcription factor, TFEB (Transcription factor EB), and its related autophagic target genes. In particular, TFEB expression was determined in HCC cells after treatment with 100 nM panobinostat. An induction of TFEB in HepG2 and Hep3B cells was observed after 48 h of treatment. The transcript increased steadily up to 72 h. (Figure 1A). Furthermore, an increase in the expression of all analyzed autophagic markers was observed. The levels of ATG12 and TP73 were below the control level in HepG2 cells (Figure 1B). TP73 does not exert any role in HepG2 cells as they have wild type TP53, which is stably expressed and plays a key role in these cells as previously published [18].

**Figure 2:**
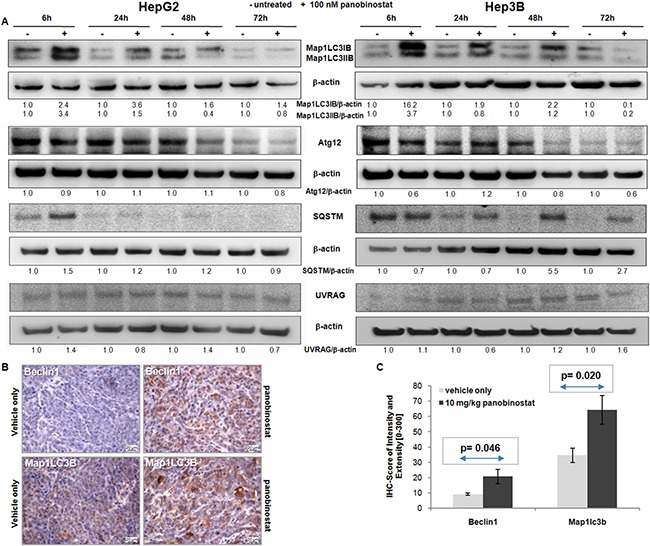
Autophagic protein modulation (**A**) Map1LC3B, Atg12, Sqstm and UVRAG protein level was determined in HepG2 and Hep3B cells after 72 h of treatment with 100 nM panobinostat. Densitometry results were normalized to β-actin content and are expressed relative to untreated controls set at 1.0. (**B**) HepG2 xenografts originated in NMRI mice. Mice were treated for 4 weeks with 10 mg/kg panobinostat. Beclin1 and Map1LC3B were detected by immunohistochemistry in the cytosol. Nuclei were counterstained with hematoxylin. Magnification is 400× and scale bar represents 20 μm. (**C**) Immune reactivity score ± S.E.M. of Beclin1 and Map1LC3B, based on the score of intensity (0–300) and percentage of stained cells. *P* < 0.05 was regarded as significant.

Semi-quantitative western blot of autophagic markers was performed in HepG2 and Hep3B cells after treatment with 100 nM panobinostat. As shown in Figure 2A, panobinostat caused a strong increase in Map1LC3B protein level already after 6 h. In particular, a strong up-regulation of the lipidated form of Map1LC3B was detected; which can be visualized as the lowest band on the membrane. Its level decreased in Hep3B cells only after 72 h treatment. Sqstm, a gold standard autophagic marker, was also up-regulated in HepG2 cells after 6 h and in Hep3B cells after 48 h. The expression of Atg12 and UVRAG was unchanged in both cell lines, thus supporting their involvement in the autophagosome formation.

**Figure 3:**
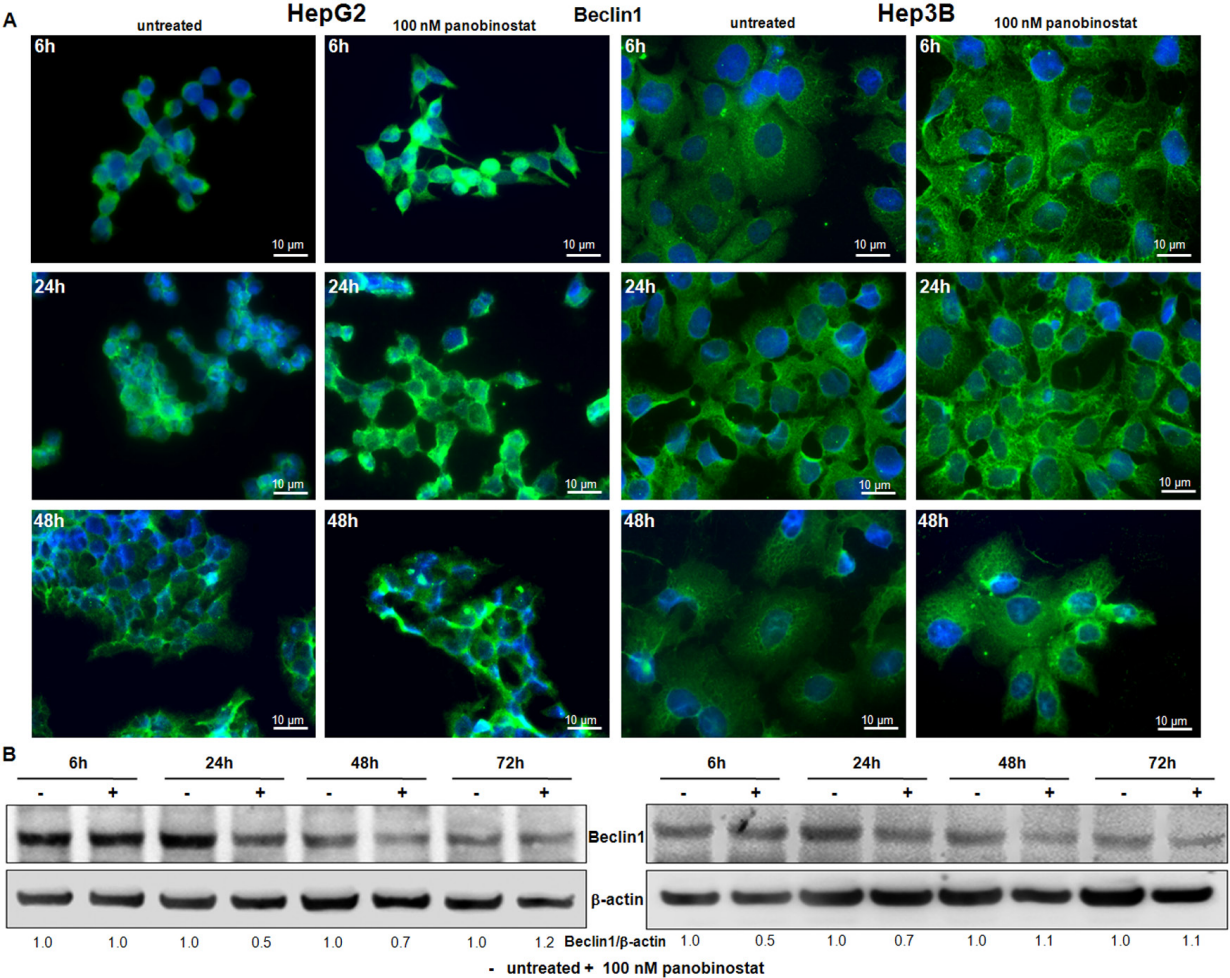
Beclin1 involvement (**A**) Beclin1 protein distribution was determined by immunofluorescence in HepG2 and Hep3B cells. After treatment with 100 nM panobinostat, Beclin1 aggregates are clearly visible. Immunofluorescence analysis has been performed under identical settings. Nuclei were stained with Hoechst 33342. Magnification is 630× and scale bar represents 10 μm. (**B**) Western blot of Beclin1. Densitometry results were normalized to β-actin content and are expressed relative to untreated controls set at 1.0.

Beclin1 and Map1LC3B expression was analyzed by immunohistochemistry in HepG2 tumor xenografts in mice that had been treated for 4 weeks with 10 mg/kg panobinostat [18]. As shown in Figure 2B, treatment with panobinostat caused a massive increase in the expression of Beclin1 and Map1LC3B in HepG2 tumor xenografts. These markers were expressed at basal level in xenografts treated with vehicle only. Beclin1 and Map1LC3B are located in the cytosolic subcellular compartments. Expression of these markers was quantified based on IRS and showed a significant (*P* < 0.05) up-regulation after panobinostat intraperitoneal treatment (Figure 3B). The *in vivo* up-regulation of these key players strongly supports the proposed autophagic model for HCC cell lines *in vitro*.

### Analysis of Beclin1 subcellular distribution in liver cancer cells

Beclin1 expression is used to detect the autophagosome formation based on its subcellular distribution. For this reason, immunofluorescent staining for beclin1 was performed in HepG2 and Hep3B cells after 24 h and 48 h of incubation with 100 nM panobinostat. As shown in Figure 3A, beclin1 had an aggregated structure in cells treated with panobinostat, as confirmed by green fluorescent spotting. Panobinostat treatment did not cause any change in beclin1 levels as determined by western blot (Figure 3B).

### P53 family involvement in the autophagic process induced by panobinostat

**Figure 4:**
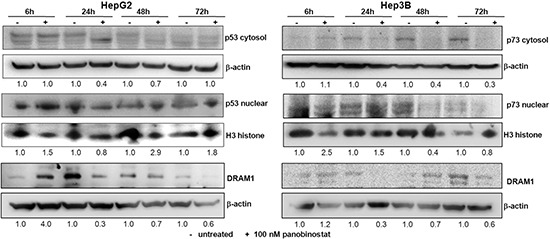
p53 and p73 role during autophagy P53 (HepG2) and p73 (Hep3B) protein levels were detected in cytosol and nuclear subcellular fractions after treatment with 100 nM panobinostat. DRAM1 was detected in whole cell lysates. Densitometry results were normalized to β-actin (cytosol) and Histone H3 (nuclei) content and are expressed relative to untreated controls set at 1.0.

The ability of p53 to control autophagic processing is dependent on its subcellular localization. In particular, DRAM1 (DNA damage regulated autophagy modulator 1) is a transcriptional target of nuclear p53 and promotes autophagic cell death [23, 24]. This study focused on dissecting the role of p53 in HepG2 cells and its related p73 in Hep3B cells. Cytosolic levels of p53 and p73 were lowered drastically by treatment with 100 nM panobinostat and a strong nuclear accumulation was detected already after 6 h. Furthermore, it could be proven that DRAM1, the autophagic marker transcriptionally regulated by p53 family members, was up-regulated in HepG2 after only 6 h; whereas its level was stable in Hep3B cells also after 6 h (Figure 4). Its level was down-regulated in both cell lines after 24 h due to its role in autophagosome vesicle formation. The nuclear localization of p53 and p73 is required for their role in pro-death autophagic activity.

### Autophagosome dynamic in liver cancer cells under the effect of panobinostat

**Figure 5:**
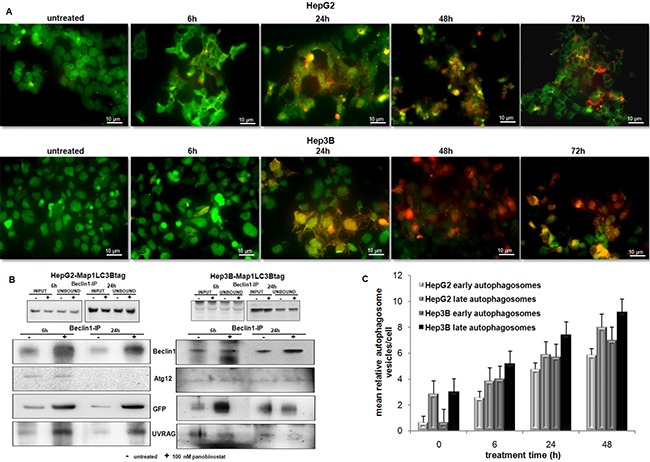
Autophagosome dynamic and aggregation (**A**) Dynamic of autophagosomes in HepG2 and Hep3B cells stably transfected with MAP1LC3B-GFP-RFP-tag. Treatment with 100 nM panobinostat (6 to 72 h) caused a time dependent shift of fluorescence. RFP lightening appeared stronger than green fluorescence at late treatment time. Control cells are shown at time zero of treatment. Panobinostat caused massive cell death after 72 h. (**B**) Immunoprecipitation of beclin1 in HepG2 and Hep3B after a short time of treatment. Autophagosomes components Atg12, Map1LC3B and UVRAG increased after treatment with 100 nM panobinostat. Ectopic Map1LC3B (GFP) was detected. (**C**) Quantification of early and late autophagosome vesicles detected by T.E.M. Mean relative amount of vesicles in HepG2 and Hep3B cells ± SD is shown.

In order to follow the dynamic of autophagosome formation and dissect each step of the maturation process of autophagosomal vesicles, HepG2 and Hep3B cells were stably transfected with a plasmid containing MAP1LC3-GFP-RFPtag [25]. After the selection, the stable clones were treated with 100 nM panobinostat for 72 h (Figure 5A). The green fluorescence, a marker of early stage of autophagosome maturation, was clearly visible up to 6 h in both cell lines; the red fluorescence intensity increased after 24 h of treatment, confirming the maturation process of the autophagosome. The green fluorescence decreased due to the degradation of GFP during the fusion of the autophagosomes with the lysosomes. These results suggest that autophagy is not blocked aberrantly in this proposed model and that it represents an active process in HCC cells treated with panobinostat.

Furthermore, the formation of autophagosome was proven by immunoprecipitation of its key factors in HepG2 and Hep3B cells (Figure 5B). Beclin1 was expressed strongly after 6 h of treatment with 100 nM panobinostat in both cell lines. Analysis of beclin1 precipitate identified Atg12, the ectopic Map1LC3B and UVRAG as shown in Figure 5B. Their amount was obviously higher in the protein precipitate of treated cells than of control cells. Panobinostat triggered an accumulation of the complex formed by Beclin1, Atg12, Map1LC3B and UVRAG, underlying the ongoing nucleation step of autophagosomes.

### Transmission electron microscopy analysis of autophagy

**Figure 6:**
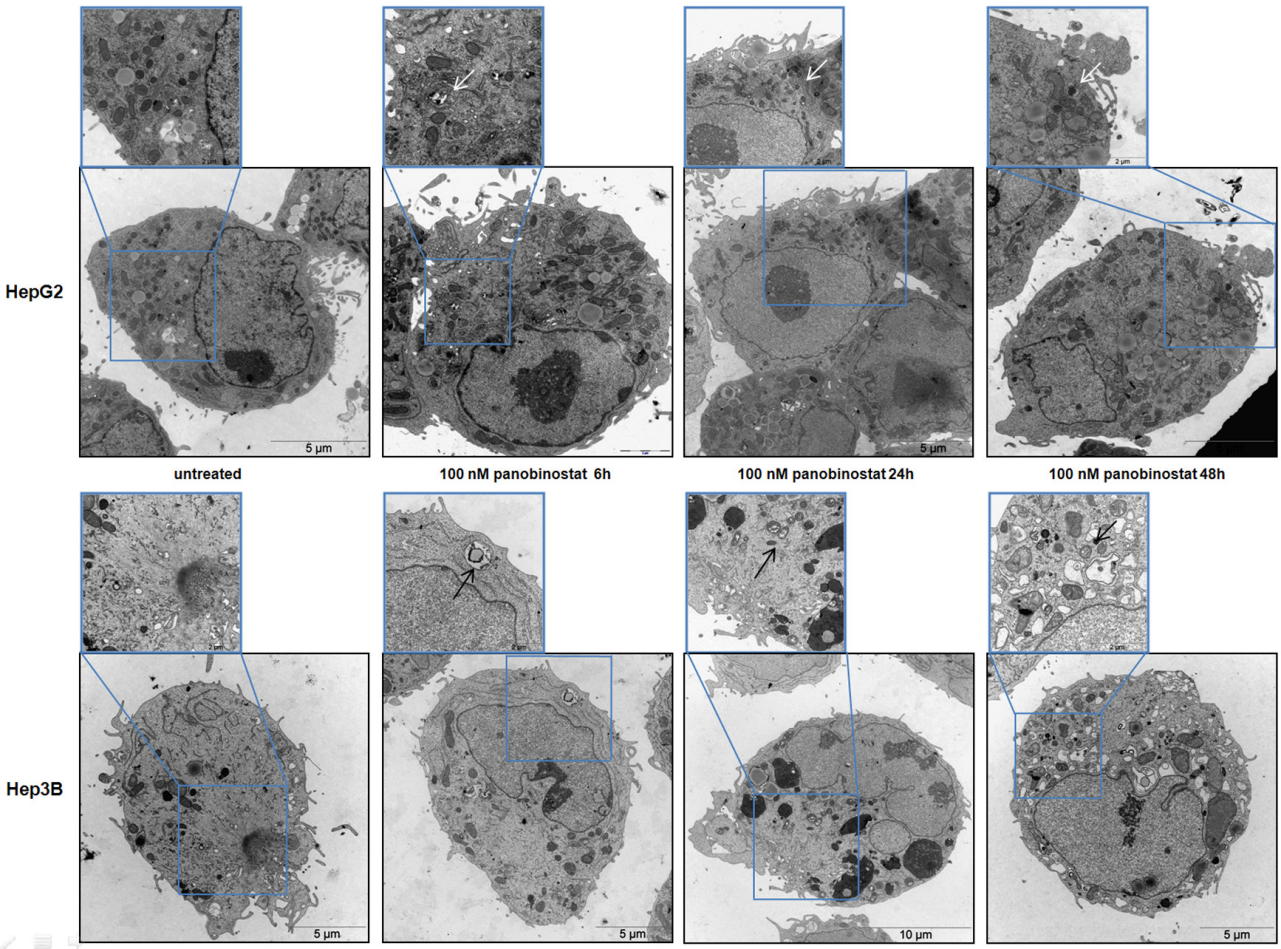
HCC cells ultrastructure analysis HepG2 (upper panels) and Hep3B (low panels) cells were incubated with 100 nM panobinostat for 48 h. The formation of double membrane vesicles and a disorganized endoplasmic reticulum are visible. This pattern is magnified in the insets that show an enlargement of each picture. Magnification is 2156 and 35,570 X and scale bars represent 5 and 10 μm.

Additionally, the autophagosome vesicles in cells undergoing the autophagic process could be detected by transmission electron microscopy. In Figure 6, qualitative ultrastructural analysis shows the formation of double-layered subcellular vesicles after treatment with 100 nM panobinostat in HepG2 and Hep3B cells. The autophagosomes, indicated in Figure 6 by white and black arrows, are clearly visible already after 6 h and the amount increased after 48 h, especially in Hep3B cells. In addition, as previously published, the micrographs show a disorganized endoplasmic reticulum with fragmented cisternae representing a clear sign of active autophagy [19]. Based on a previously published method [26, 27], the number of vesicles was quantified from around 100 micrographs and divided into two subgroups: early autophagosomes characterized by a double membrane and late autophagosomes as fused with endo/lysosomal vesicles including subcellular particles (Figure 5C). Statistical analysis was performed, as shown in Supplementary Table S1, thus confirming that the number of early and late autophagosomes after treatment with 100 nM panobinostat increased significantly in both cell lines.

### Real time cell viability measured during treatment with autophagy modulators

**Figure 7:**
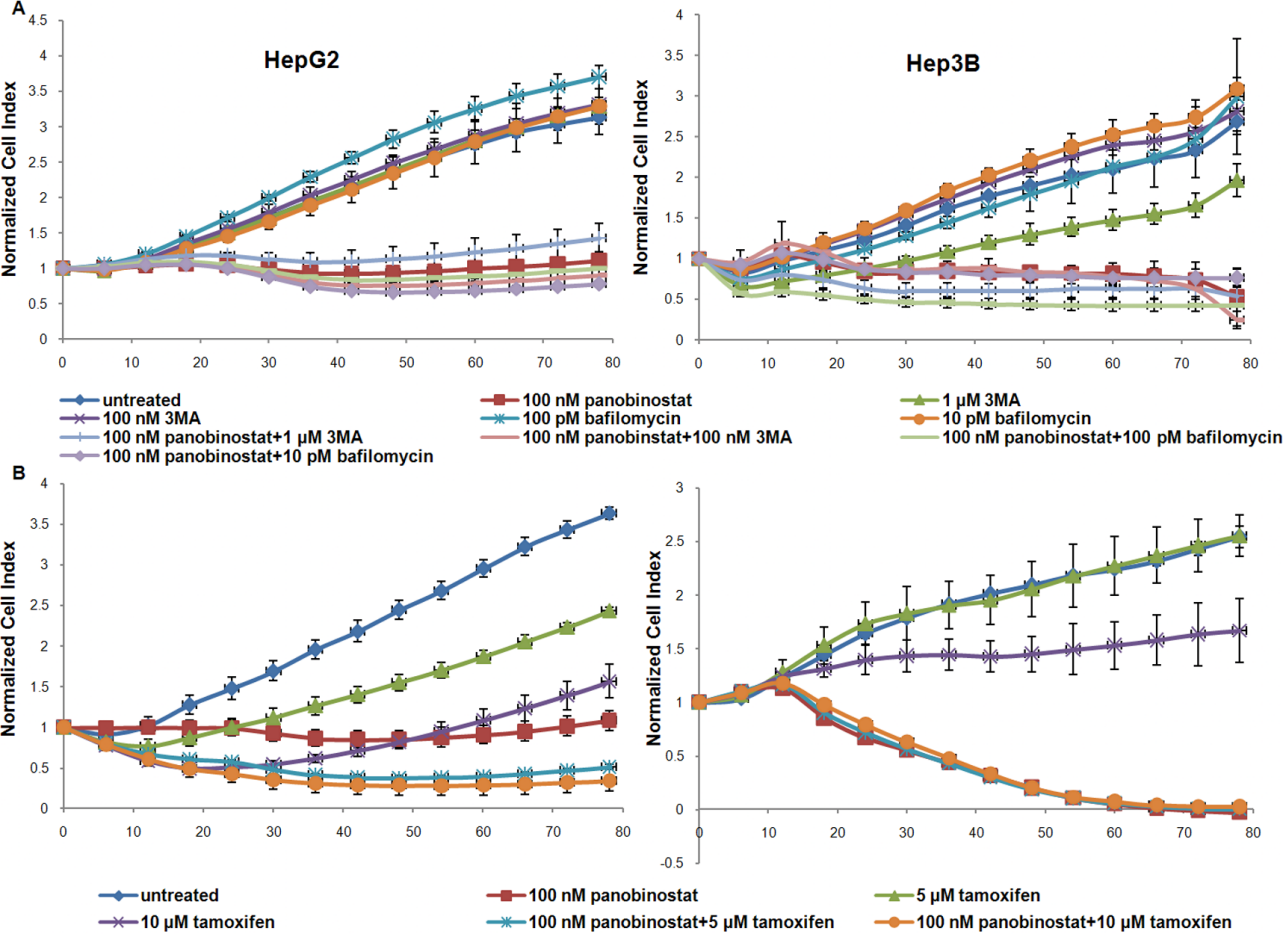
Autophagy mediated cell viability (**A**) HepG2 and Hep3B cells were cultured in E-plates and, after approx. 24 h, treated with 100 nM panobinostat, 1 μM and 100 nM 3MA, 100 and 10 pM Bafilomycin. In panel (**B**) HCC cells were incubated with 5 and 10 μM Tamoxifen alone and in combination with 100 nM panobinostat. Cell index was normalized to the time point of treatment. Cell index was determined continuously for additional 80 h. Shown are means ± SD of three independent experiments performed in triplicates.

In order to finally confirm the contribution of autophagy to cell demise, HCC cells were incubated with autophagy modulators. Cell viability was measured by impedance based real time cell viability device xCelligence. The HepG2 and Hep3B cell lines were incubated with 3-methyl-adenine (3MA) and Bafilomycin at different concentrations. As shown in Figure 7A the block of autophagy caused by 3MA and Bafilomycin did not cause any reduction in cell viability and 1 μM 3MA caused a slight reduction in cell viability only in Hep3B cells. Autophagy blockade alone could not prevent panobinostat efficacy due to its broad spectrum of action [18–21, 28]. However, treatment with the autophagy inducer, Tamoxifen, led to a strong reduction in cell viability in both HepG2 and Hep3B cell lines. Furthermore, Tamoxifen showed an additive effect in combination with 100 nM panobinostat as seen by the decrease of the viability curve in the HepG2 cell line.

In conclusion, autophagy sustains panobinostat-mediated cell death in concert with the other ongoing mechanisms previously described [18–21].

## DISCUSSION

Hepatocellular carcinoma is a cancer with a high rate of incidence worldwide. Currently, the HCC rate in western countries is increasing and this tumor disease still has a poor prognosis. It is the third most common cause for cancer death in the world. Therapy for HCC is still inefficacious. The tyrosine kinase inhibitor, Sorafenib, despite its limitations, such as low overall response rates and high toxicity, was the first systemic targeted therapy approved by the US Food and Drug Administration for the treatment of advanced HCC [29].

Recent discoveries have shown that deacetylase inhibitors (DACi) possess a broad spectrum of activities in cancer cells. DACi are currently approved for the treatment of lymphoma and are in clinical trials for several solid and hematologic malignancies. SAHA (vorinostat, zolinza) is FDA approved in combination with Trichostatin A. Panobinostat, a novel pan-deacetylase inhibitor with a cinnamic hydroxamic structure, is currently under approval for treatment of patients affected by lymphoma. It has already been shown that, despite their specific inhibition of all classes of HDACs, DACi can promote the hyperacetylation of p53 and HSP90, thus modulating the cell demise program [17].

Additionally, panobinostat was shown to be able to induce the activation of non-canonical apoptotic pathways; its activity could promote cell demise *via* endoplasmic reticulum stress [18, 19]. Furthermore, panobinostat is responsible for the up-regulation of hsa-let-7b, a tumor suppressor miRNA, and the consequent suppression of its target HMGA2 [20]. Oncogenic miRNAs belonging to the miR17-92 cluster are suppressed after treatment with panobinostat in liver cancer cells, which strongly supports its broad spectrum of activity that leads to cell demise in the proposed model [21]. In liver cancer cells treatment with panobinostat also influences their epithelial mesenchymal transition status [30].

Since its discovery, autophagy has been shown to play a pivotal role in the cell [31]. It is a conserved mechanism that is involved in physiology, development and lifespan, which leads to the degradation of proteins and organelles accumulating within the cytosolic compartments of the cell [32]. Moreover, prolonged autophagy can contribute to survival during metabolic stress, e.g. nutrient deprivation, and also can provoke cell death [32]. Its role as a double-edged sword is not yet fully elucidated, but is involved in tumorigenesis, leading to survival or cell demise [33]. Autophagy represents a clearance mechanism activated by liver cells during stress, e.g. obesity, alcohol consumption, hepatitis infections. Its aberrant function leads to accumulation of fatty acids, viral particles and provokes liver cell death, cirrhosis and tumorigenesis [34]. In addition, inhibition or impairment of autophagy confers resistance to autophagy-related cell death in HCC cells [35].

Autophagy as a potential therapeutic target mechanism has been of interest in liver oncology for the last few years, especially for the treatment of HCC at a metastatic stage, where the admission of alternative compounds for clinical trials is favored [36].

Here we dissected the autophagy mechanism acting in liver cancer cells after treatment with the deacetylase inhibitor, panobinostat. Initially, the components of autophagic vesicles were analyzed in detail according to previously published guidelines [22]. Interestingly, all analyzed autophagy markers were stable and/or up-regulated in liver cancer cells together with their attributed transcription factor, TFEB, as has been described previously [37]. Liver cancer cells showed a basal expression of autophagy markers that are up-regulated after panobinostat treatment, proving that panobinostat exerts a pro-autophagy activity in the proposed model, and further clarifies its role in combination with Sorafenib [38]. SAHA has been shown to have similar effects in liver cancer cells, but only at higher concentrations [39].

Despite its stable expression *in vitro*, the cellular distribution of beclin1 changed after treatment in both cell lines and its spotted appearance confirmed its involvement in autophagosome formation. Beclin1 is a structural protein that, together with Map1LC3B and other autophagy proteins, composes the autophagosomes vesicles during their nucleation step [40]. Furthermore, beclin1 is essential during embryonal development and is a haploinsufficient tumor suppressor [41]. Its loss results in a high rate of lymphoma, lung and liver cancer in mice. Its heterozygosity, together with HBV large envelope protein expression, increases liver tumor incidence [8]. Interestingly, it was observed here that beclin1 expression was up-regulated in tumor xenografts in mice, thus supporting that its involvement is triggered by deacetylase inhibitors in the proposed model.

P53 and its family members play a Janus role during autophagy. Despite its well known role as guardian of the cell fate through the control of apoptotic pathway, p53 exerts a double-edged role during autophagy by promoting a pro-survival or a pro-death autophagic machinery [23]. In particular, DRAM1 is a transcriptional target of nuclear p53 and promotes autophagic cell death [42]. Cytosolic p53 seems to promote a survival autophagy in the absence of its pro-death targets [23] and/or its regulator hdm2 [43]. Here, panobinostat caused a down-regulation of the cytosolic p53 and its nuclear up-regulation followed by a stable level of its target DRAM1. Thus, nuclear p53, together with p73, participates actively in the cell demise induced by panobinostat through autophagy.

Map1LC3B, a structural component of autophagosomes, was up-regulated by panobinostat in liver cancer cells and in mice xenografts. Treatment caused an increase of the cleaved and lipidated form of Map1LC3B confirming its role during autophagosomal nucleation phase as previously published [44]. Additionally, stable transfection with GFP-RFP-Map1LC3B revealed that panobinostat promoted the maturation of autophagosomes in HCC cells. Ectopic Map1LC3B forms a stable complex with Beclin1, UVRAG and Atg12 that increased significantly after treatment. This protein, together with other atg proteins, constitutes the backbone of the autophagosomes and contributes to the final fusion with the lysosome [45]. HCC cells underwent vesicle budding after treatment with panobinostat. Double membrane vesicles were observed in HepG2 and Hep3B cells already after treatment for a short time, which can be attributed to autophagosomes formation [22]. In line with these findings, ER-stress caused by panobinostat, followed by massive degradation of endoplasmic reticulum, contributes to autophagosome assembly [19]. Finally, blocking of autophagy with 3MA or Bafilomycin increased cell viability of HepG2 and Hep3B cells, whereas its activation with Tamoxifen, a positive control for autophagy, lowered cell viability and increased the efficacy of panobinostat. Great hopes were set on Tamoxifen by triggering estrogen receptor system in HCC resulting in an inhibition of tumor growth. Unfortunately, the clinical trials were disappointing due to the absence of clinical benefit. Despite the modest side effects of Tamoxifen, no significant survival prolongation was observed for patients suffering from HCC [46]. Interestingly, beside the effects on the estrogen receptor system, Tamoxifen is a strong inducer of autophagy [47, 48]. Hence, in combination with panobinostat, it boosts autophagy to overcome a threshold leading to apoptosis.

Finally, as previously published, panobinostat caused a down-regulation of the survival factor ERK (MAPK1, Mitogen-Activated Protein Kinase 1) [18], which could not interfere with autophagy induction as proven by Tong Y. et al. [49]. Induction of autophagy provokes cell death in HCC cells, confirming the previously described mechanism in glioma, liver and breast cancer [38, 50, 51]. Furthermore, its activity in the proposed model could be responsible for the degradation of oncomiRNAs restoring the expression of pro-apoptotic factors as previously published [52].

In conclusion, panobinostat leads to autophagy in HCC cells and combines with a broad spectrum of mechanisms to induce cell demise. The liver cancer cells used for this study present a basal expression of autophagic players that trigger cell death under the control of panobinostat. Currently, autophagy-mediated cell death represents a new strategy for cancer treatment, especially for tumors that develop resistance to apoptosis. The findings described here give an overview of the broad activity of deacetylase inhibitors and support future therapeutic strategies for solid tumors. Furthermore, the multimodal combination of previously approved clinical compounds such as tamoxifen and panobinostat, with its pleiotropic effects in addition to the induction of autophagy, could lead to an urgently needed systemic anticancer strategy for HCC.

## MATERIALS AND METHODS

### Cell culture and reagents

Human HCC cell lines, HepG2 (TP53wt) (ACC-180) and Hep3B (TP53null) (ACC-93) (DSMZ, Deutsche Sammlung von Mikroorganismen und Zellkulturen, Braunschweig Germany) were cultured under standard conditions as previously described [18]. Panobinostat was kindly provided by Novartis Pharma AG (Basel, Switzerland) and prepared as previously described [18]. 4-Hydroxytamoxifen was a kind gift from Heidi Griesmann (Department of Gastroenterology and Endocrinology, Philipps University of Marburg). 3-methyladenine (M9281) and Bafilomycin (B1793) were purchased from Sigma-Aldrich.

### Xenograft model of hepatocellular carcinoma

Paraffin blocks were obtained from a previous study of a xenograft model of HCC [18].

All animals received human care. The study protocol complied with the institute's guidelines and was approved by the Government of Lower Franconia (Würzburg, Germany, file number 54-2531.31-3/06) before beginning the experiments. Hep3B cells proved not to be tumorigenic in NMRI mice and were therefore not used for *in vivo* experiments.

### Quantitative RT-PCR

For quantitative real time PCR, total RNA was extracted using the RNeasy Mini Kit (QIAGEN, 74106) according to the manufacturer's instructions and reverse transcription (RT) was performed with Quantitect Reverse Transcription Kit (QIAGEN, 205311). Primers for human BECN1 (QT00004221), MAP1LC3B (QT00055069), UVRAG (QT00034328), ATG5 (QT00073325), ATG12 (QT00035854), AMBRA1 (QT00093303), TFEB (QT00069951), TP73 (QT00030240), P62 (SQSTM1) (QT00095676) and GAPDH (QT01192646) were purchased from QIAGEN and run with SsoFast^™^ EvaGreen^®^ Supermix (BIORAD, #172-5200) on a CFX96 Real Time PCR Detection System (BIORAD). Results were analyzed with the CFX Manager v2.0 and Rest 2008 software and normalized to GAPDH mRNA content for each sample.

### Protein extraction and western blot analysis

Whole cell lysates were further processed by SDS-Page followed by Western blotting, as previously described [18]. Immunodetection with primary antibodies against Map1LC3B (ab51520), Atg12 (ab56465) Sqstm1 (ab96706), Beclin1 (ab114071) (AbCam), UVRAG (U7508) and b-actin (A5441) (Sigma-Aldrich) was performed. Bound secondary HRP-conjugated anti-mouse (A9917) and anti-rabbit (A0545) antibodies (Sigma-Aldrich) were detected by incubating the immunoblots with SuperSignal West Pico Chemiluminescent Substrate (#34077, Pierce, Thermo Fisher Scientific). The luminescent reactivity was then measured using Fusion image capture and further quantified with Bio1D analysis system (PEQLAB Biotechnologie GmbH). Anti-b-actin was used to control equal loading and protein quality.

### Immunohistochemistry (IHC)

Paraffin-embedded xenograft tumor samples from previous experiments [18, 20] were cut into 5 mm sections deparaffinized using graded alcohols. Antigen retrieval was performed by heat-induced epitope retrieval in pH 9 antigen retrieval buffer (Dako, Glostrup, Denmark) at 95°C for 60 min. Endogenous peroxidase blocking was carried out for 10 min with peroxidase blocking reagent (Dako). Subsequently, the primary antibody for beclin1 (mouse monoclonal ab114071, 1:500) and Map1LC3B (rabbit polyclonal, ab51520, 1:500) was applied for 30 min at RT. Additionally, the mouse or rabbit linker (Dako) was used for signal enhancement. Detection and visualization was performed by using the Envision^™^ Flex Kit (Dako) based on diaminobenzidine (DAB) as the chromogen substrate (Roche Molecular Biochemicals, Mannheim, Germany) according to the manufacturer's instructions. Slides were counterstained with hematoxylin. Immunohistochemical staining was performed on the platform Autostainer Plus (Dako). The stained slides were digitalized using the ImageAccess 9 Enterprise software (Imagic Bildverarbeitung, Glattbrugg, Switzerland). Afterwards, an immune reactivity score (IRS; 0–300) was calculated by multiplying the scores for intensity (0–3) and the stained cells (0–100%) as previously published [53].

### Immunofluorescence

HepG2 and Hep3B cells were seeded in chamber slides (Lab-Tek distributed by Fisher Scientific, 177399) and Immunofluorescence was performed as previously described [20]. Primary antibody against Beclin1 (ab114071) and secondary AlexaFluor488-conjugated antibody (A11001) from Life Technologies were used. The fluorescence was visualized with a Nikon microscope at 630X magnification and acquired with a Hamamatsu ORCA-ER camera (C4742-80) under the same setting. The acquired data were analyzed with ImageJ software v 1.43u.

### Cytosolic and nuclear fraction

Subcellular fractions were isolated from HepG2 and Hep3B as previously described [21]. Samples were processed by western blot as previously described [20]. Primary antibodies against p53 (554294, BD Biosciences) and p73 (ab22045, AbCam) were used to detect the protein content in the nuclear and cytosolic fractions. Histone H3 (39163, Active Motif) and b-actin were used as equal loading and quality control.

### Stable transfection

The cells were incubated with a plasmid encoding for EGFP-mRFP-MAP1LC3B (*Rattus norvegicus*) (Addgene plasmid 21074) [25], at final concentration of 2 μg/ml previously mixed with serum-free, antibiotic-free Medium and FUGENE HD Transfection Reagent (Promega, E2311). After 72 hours, normal growth medium containing G418 (900 μg/ml) was added to the cells. After 7 days, the fluorescent colonies were selected and seeded in 24-well plate with 500 μg/ml G418. After 3 weeks, the single colony generated a population of stably transfected cells expressing the EGFP-mRFP-MAP1LC3B plasmid. Transfection was confirmed by western blotting and by fluorescence microscopy, as previously described.

### Immunoprecipitation

HepG2 and Hep3B cells were treated for 6 and 24 hours with 100 nM panobinostat. Immunoprecipitation was performed by the use of Pierce^®^ Crosslink Immunoprecipitation Kit (Thermo Scientific, 26147). Beclin1 was precipitated with the antibody ab114071 (AbCam). The final elutes were processed by western blotting to detect Beclin1, UVRAG, Atg12 as described above. Ectopic EGFP-mRFP-MAP1LC3B was detected with a primary antibody for GFP (ab1218) from AbCam.

### Sample processing for transmission electron microscopy (T.E.M.)

HepG2 and Hep3B cells were treated with panobinostat for 48 h. Cells were processed as previously described [19]. Quantification of autophagic vacuoles [26, 27] was performed on around 100 cells from T.E.M. sections prepared from untreated and 100 nM panobinostat treated HepG2 and Hep3B cells. Characteristic double membrane autophagosomes were counted as initial/early autophagic vacuoles whereas autophagosomes that had fused with vesicles originated from the endo/lysosomal compartment were counted as degradative/late autophagic vacuoles. Data represent mean vesicle number per cell ± SD (Figure 5C and Supplementary Table S1).

### Real-time cell viability analysis

The xCELLigence RTCA SP system (Roche Applied Science) was used, for real-time analysis of the cellular response of HepG2 and Hep3B cells following incubation with 100 nM panobinostat, 5–10 μM 4-HidroxyTamoxifen, 100 nM 1 μM 3-methyladenine and 10–100 pM Bafilomycin as previously described [19–21]. Cell index, indicating attachment and adherence of cells to the plate's electrode, was measured continuously for the following 80 hours. Data analysis was performed using the RTCA Software v1.2.1.

### Statistical analysis

Statistical analysis was performed using SPSS 15.0.1 for Windows (SPSS Inc., Chicago, IL, USA). Significance was calculated using the *t*-test for paired samples and ANOVA-Bonferroni post-Hoc test. **p < 0.01 and *p < 0.05 were regarded as significant.

## SUPPLEMENTARY MATERIALS TABLE



## References

[1] Bosch FX, Ribes J, Diaz M, Cleries R (2004). Primary liver cancer: worldwide incidence and trends. Gastroenterology.

[2] Simonetti RG, Liberati A, Angiolini C, Pagliaro L (1997). Treatment of hepatocellular carcinoma: a systematic review of randomized controlled trials. Ann Oncol.

[3] Denton D, Xu T, Kumar S (2015). Autophagy as a pro-death pathway. Immunol Cell Biol.

[4] Levine B, Kroemer G (2008). Autophagy in the pathogenesis of disease. Cell.

[5] Gray JV, Petsko GA, Johnston GC, Ringe D, Singer RA, Werner-Washburne M (2004). “Sleeping beauty”: quiescence in Saccharomyces cerevisiae. Microbiol Mol Biol Rev.

[6] Liang C, Jung JU (2010). Autophagy genes as tumor suppressors. Curr Opin Cell Biol.

[7] Kroemer G, Levine B (2008). Autophagic cell death: the story of a misnomer. Nat Rev Mol Cell Biol.

[8] Qu X, Yu J, Bhagat G, Furuya N, Hibshoosh H, Troxel A, Rosen J, Eskelinen EL, Mizushima N, Ohsumi Y, Cattoretti G, Levine B (2003). Promotion of tumorigenesis by heterozygous disruption of the beclin 1 autophagy gene. J Clin Invest.

[9] Fischer TD, Wang JH, Vlada A, Kim JS, Behrns KE (2014). Role of autophagy in differential sensitivity of hepatocarcinoma cells to sorafenib. World J Hepatol.

[10] Liang C, Feng P, Ku B, Dotan I, Canaani D, Oh BH, Jung JU (2006). Autophagic and tumour suppressor activity of a novel Beclin1-binding protein UVRAG. Nat Cell Biol.

[11] Ruivo R, Anne C, Sagne C, Gasnier B (2009). Molecular and cellular basis of lysosomal transmembrane protein dysfunction. Biochim Biophys Acta.

[12] Laurin N, Brown JP, Morissette J, Raymond V (2002). Recurrent mutation of the gene encoding sequestosome 1 (SQSTM1/p62) in Paget disease of bone. Am J Hum Genet.

[13] Hale AN, Ledbetter DJ, Gawriluk TR, Rucker EB (2013). Autophagy: regulation and role in development. Autophagy.

[14] Loos B, Engelbrecht AM, Lockshin RA, Klionsky DJ, Zakeri Z (2013). The variability of autophagy and cell death susceptibility: Unanswered questions. Autophagy.

[15] Mah LY, Ryan KM (2012). Autophagy and cancer. Cold Spring Harb Perspect Biol.

[16] Gryder BE, Sodji QH, Oyelere AK (2012). Targeted cancer therapy: giving histone deacetylase inhibitors all they need to succeed. Future Med Chem.

[17] Anne M, Sammartino D, Barginear MF, Budman D (2013). Profile of panobinostat and its potential for treatment in solid tumors: an update. Onco Targets Ther.

[18] Di Fazio P, Schneider-Stock R, Neureiter D, Okamoto K, Wissniowski T, Gahr S, Quint K, Meissnitzer M, Alinger B, Montalbano R, Sass G, Hohenstein B, Hahn EG (2010). The pan-deacetylase inhibitor panobinostat inhibits growth of hepatocellular carcinoma models by alternative pathways of apoptosis. Cell Oncol.

[19] Montalbano R, Waldegger P, Quint K, Jabari S, Neureiter D, Illig R, Ocker M, Di Fazio P (2013). Endoplasmic reticulum stress plays a pivotal role in cell death mediated by the pan-deacetylase inhibitor panobinostat in human hepatocellular cancer cells. Transl Oncol.

[20] Di Fazio P, Montalbano R, Neureiter D, Alinger B, Schmidt A, Merkel AL, Quint K, Ocker M (2012). Downregulation of HMGA2 by the pan-deacetylase inhibitor panobinostat is dependent on hsa-let-7b expression in liver cancer cell lines. Exp Cell Res.

[21] Henrici A, Montalbano R, Neureiter D, Krause M, Stiewe T, Slater EP, Quint K, Ocker M, Di Fazio P (2015). The pan-deacetylase inhibitor panobinostat suppresses the expression of oncogenic miRNAs in hepatocellular carcinoma cell lines. Mol Carcinog.

[22] Klionsky DJ, Abdalla FC, Abeliovich H, Abraham RT, Acevedo-Arozena A, Adeli K, Agholme L, Agnello M, Agostinis P, Aguirre-Ghiso JA, Ahn HJ, Ait-Mohamed O, Ait-Si-Ali S (2012). Guidelines for the use and interpretation of assays for monitoring autophagy. Autophagy.

[23] Ryan KM (2011). p53 and autophagy in cancer: guardian of the genome meets guardian of the proteome. Eur J Cancer.

[24] Levine B, Abrams J (2008). p53: The Janus of autophagy?. Nat Cell Biol.

[25] Kimura S, Noda T, Yoshimori T (2007). Dissection of the autophagosome maturation process by a novel reporter protein, tandem fluorescent-tagged LC3. Autophagy.

[26] Mitou G, Frentzel J, Desquesnes A, Le Gonidec S, AlSaati T, Beau I, Lamant L, Meggetto F, Espinos E, Codogno P, Brousset P, Giuriato S (2015). Targeting autophagy enhances the anti-tumoral action of crizotinib in ALK-positive anaplastic large cell lymphoma. Oncotarget.

[27] Barth S, Glick D, Macleod KF (2010). Autophagy: assays and artifacts. J Pathol.

[28] Di Fazio P, Montalbano R, Quint K, Alinger B, Kemmerling R, Kiesslich T, Ocker M, Neureiter D (2013). The pan-deacetylase inhibitor panobinostat modulates the expression of epithelial-mesenchymal transition markers in hepatocellular carcinoma models. Oncol Lett.

[29] Murata S, Mine T, Sugihara F, Yasui D, Yamaguchi H, Ueda T, Onozawa S, Kumita SI (2014). Interventional treatment for unresectable hepatocellular carcinoma. World J Gastroenterol.

[30] P DIF, Montalbano R, Quint K, Alinger B, Kemmerling R, Kiesslich T, Ocker M, Neureiter D (2013). The pan-deacetylase inhibitor panobinostat modulates the expression of epithelial-mesenchymal transition markers in hepatocellular carcinoma models. Oncol Lett.

[31] Deter RL (1971). Quantitative characterization of dense body, autophagic vacuole, and acid phosphatase-bearing particle populations during the early phases of glucagon-induced autophagy in rat liver. J Cell Biol.

[32] Yang Z, Klionsky DJ (2010). Eaten alive: a history of macroautophagy. Nat Cell Biol.

[33] Tian Y, Kuo C, Sir D, Wang L, Govindarajan S, Petrovic LM, Ou JJ (2015). Autophagy inhibits oxidative stress and tumor suppressors to exert its dual effect on hepatocarcinogenesis. Cell Death Differ.

[34] Rautou PE, Mansouri A, Lebrec D, Durand F, Valla D, Moreau R (2010). Autophagy in liver diseases. J Hepatol.

[35] Huang G, Jiang Q, Cai C, Qu M, Shen W (2015). SCD1 negatively regulates autophagy-induced cell death in human hepatocellular carcinoma through inactivation of the AMPK signaling pathway. Cancer Lett.

[36] Peng YF, Shi YH, Ding ZB, Ke AW, Gu CY, Hui B, Zhou J, Qiu SJ, Dai Z, Fan J (2013). Autophagy inhibition suppresses pulmonary metastasis of HCC in mice via impairing anoikis resistance and colonization of HCC cells. Autophagy.

[37] Song W, Wang F, Lotfi P, Sardiello M, Segatori L (2014). 2-Hydroxypropyl-beta-cyclodextrin promotes transcription factor EB-mediated activation of autophagy: implications for therapy. J Biol Chem.

[38] Lachenmayer A, Toffanin S, Cabellos L, Alsinet C, Hoshida Y, Villanueva A, Minguez B, Tsai HW, Ward SC, Thung S, Friedman SL, Llovet JM (2012). Combination therapy for hepatocellular carcinoma: additive preclinical efficacy of the HDAC inhibitor panobinostat with sorafenib. J Hepatol.

[39] Liu YL, Yang PM, Shun CT, Wu MS, Weng JR, Chen CC (2010). Autophagy potentiates the anti-cancer effects of the histone deacetylase inhibitors in hepatocellular carcinoma. Autophagy.

[40] Kang R, Zeh HJ, Lotze MT, Tang D (2011). The Beclin 1 network regulates autophagy and apoptosis. Cell Death Differ.

[41] Yue Z, Jin S, Yang C, Levine AJ, Heintz N (2003). Beclin 1, an autophagy gene essential for early embryonic development, is a haploinsufficient tumor suppressor. Proc Natl Acad Sci U S A.

[42] Crighton D, Wilkinson S, O'Prey J, Syed N, Smith P, Harrison PR, Gasco M, Garrone O, Crook T, Ryan KM (2006). DRAM, a p53-induced modulator of autophagy, is critical for apoptosis. Cell.

[43] Tasdemir E, Maiuri MC, Galluzzi L, Vitale I, Djavaheri-Mergny M, D'Amelio M, Criollo A, Morselli E, Zhu C, Harper F, Nannmark U, Samara C, Pinton P (2008). Regulation of autophagy by cytoplasmic p53. Nat Cell Biol.

[44] Zou M, Lu N, Hu C, Liu W, Sun Y, Wang X, You Q, Gu C, Xi T, Guo Q (2012). Beclin 1-mediated autophagy in hepatocellular carcinoma cells: implication in anticancer efficiency of oroxylin A via inhibition of mTOR signaling. Cell Signal.

[45] Wild P, McEwan DG, Dikic I (2014). The LC3 interactome at a glance. J Cell Sci.

[46] Salhab M, Canelo R (2011). An overview of evidence-based management of hepatocellular carcinoma: a meta-analysis. J Cancer Res Ther.

[47] Kohli L, Kaza N, Coric T, Byer SJ, Brossier NM, Klocke BJ, Bjornsti MA, Carroll SL, Roth KA (2013). 4-Hydroxytamoxifen induces autophagic death through K-Ras degradation. Cancer Res.

[48] Nagahara Y, Takeyoshi M, Sakemoto S, Shiina I, Nakata K, Fujimori K, Wang Y, Umeda E, Watanabe C, Uetake S, Yamori T, Dan S, Yoshimi Y (2013). Novel tamoxifen derivative Ridaifen-B induces Bcl-2 independent autophagy without estrogen receptor involvement. Biochem Biophys Res Commun.

[49] Tong Y, Huang H, Pan H (2015). Inhibition of MEK/ERK activation attenuates autophagy and potentiates pemetrexed-induced activity against HepG2 hepatocellular carcinoma cells. Biochem Biophys Res Commun.

[50] Dragowska WH, Weppler SA, Wang JC, Wong LY, Kapanen AI, Rawji JS, Warburton C, Qadir MA, Donohue E, Roberge M, Gorski SM, Gelmon KA, Bally MB (2013). Induction of autophagy is an early response to gefitinib and a potential therapeutic target in breast cancer. PLoS One.

[51] Wang YX, Xu SQ, Chen XH, Liu RS, Liang ZQ (2014). Autophagy involvement in olanzapine-mediated cytotoxic effects in human glioma cells. Asian Pac J Cancer Prev.

[52] Lan SH, Wu SY, Zuchini R, Lin XZ, Su IJ, Tsai TF, Lin YJ, Wu CT, Liu HS (2014). Autophagy-preferential degradation of MIR224 participates in hepatocellular carcinoma tumorigenesis. Autophagy.

[53] Kiesslich T, Alinger B, Wolkersdorfer GW, Ocker M, Neureiter D, Berr F (2010). Active Wnt signalling is associated with low differentiation and high proliferation in human biliary tract cancer *in vitro* and *in vivo* and is sensitive to pharmacological inhibition. Int J Oncol.

