# Supplementary material for: Autophagy-related cell death by pan-histone deacetylase inhibition in liver cancer
Source: Oncotarget. 2016 Apr 5;7(20):28998–9010. doi: 10.18632/oncotarget.8585 (PMC5045373; doi:10.18632/oncotarget.8585)
Supplement: Supplementary file 1 [file oncotarget-07-28998-s001.pdf]

Autophagy-related cell death by pan-histone deacetylase inhibition in liver cancer

Supplementary Materials

Supplementary Table S1: ANOVA-Bonferroni analysis of early and late autophagosomes counting in HepG2 and Hep3B cells treated for 48 h with 100 nM panobinostat

HepG2

**Early Autophagy**	0	6 h	24 h	48 h
0		0.000 **	0.000 **	0.000 **
6 h			0.000 **	0.000 **
24 h				0.028 *
48 h				
**Late Autophagy**	0	6 h	24 h	48 h
0		0.145	0.000 **	0.000 **
6 h			0.001 **	0.000 **
24 h				0.000 **
48 h				

Hep3B

**Early Autophagy**	0	6 h	24 h	48 h
0		0.002 **	0.000 **	0.000 **
6 h			0.038 *	0.001 **
24 h				0.184
48 h				
**Late Autophagy**	0	6 h	24 h	48 h
0		0.090	0.000 **	0.000 **
6 h			0.007 *	0.000 **
24 h				0.045 *
48 h				

**$p < 0.01$ and *$p < 0.05$ were regarded as significant.
